# Preoperative elevated *E*/*e*’ (≥ 15) with preserved ejection fraction is associated with the development of postoperative heart failure in intermediate-risk non-cardiac surgical patients

**DOI:** 10.1007/s00540-019-02728-z

**Published:** 2020-01-02

**Authors:** Midoriko Higashi, Kenji Shigematsu, Kenji Tominaga, Kazuya Murayama, Daisuke Seo, Toshikazu Tsuda, Gen Maruta, Kohei Iwashita, Ken Yamaura

**Affiliations:** 1 Department of Anesthesiology, Fukuoka University, Fukuoka, Japan; 2 Operating Rooms, Fukuoka University Hospital, Fukuoka, Japan; 3 Department of Anesthesiology and Critical Care Medicine, Kyushu University, 3-1-1 Maidashi, Higashi-ku, Fukuoka, 812-8582 Japan

**Keywords:** Cardiac function, Diastolic function, Heart failure, Pulmonary edema, Surgery

## Abstract

**Purpose:**

Left ventricular diastolic dysfunction is an independent risk factor for adverse cardiovascular morbidities and mortalities in cardiovascular and high-risk surgical patients. However, there were only a few investigations among intermediate-risk surgical patients. This study aimed to investigate postoperative heart failure (HF) in intermediate-risk surgical patients who had preoperative diastolic dysfunction with preserved ejection fraction (EF).

**Methods:**

Consecutive patients underwent intermediate-risk surgery between January 2016 and December 2018 were retrospectively evaluated. Patients with preserved EF were divided into three groups using one of the parameters of diastolic function: the ratio of early diastolic filling velocity to the peak diastolic velocity of mitral medial annulus (*E*/*e*’) ≥ 15, *E*/*e*’ between 8 and 15, and *E*/*e*’ < 8. Postoperative HF was defined as clinical symptoms and radiological evidence and low SpO_2_ less than 93%. The primary outcome was the incidence of postoperative HF and its relation to preoperative *E*/*e*’. Chi-squared test, unpaired *t* test with Welch’s correction, and multivariate logistic regression were used for analysis.

**Results:**

In total, 965 patients were included in the final analysis. Postoperative HF developed in 36/965 (3.7%) patients with preserved EF. The incidence of postoperative HF was stratified according to the *E*/*e*’, and the rates of HF occurrence in patients with *E*/*e*’ < 8, 8–15, and ≥ 15 were 1.8%, 2.7%, and 15%, respectively (*P* < 0.01).

**Conclusion:**

Preoperative elevated *E*/*e*’ (≥ 15) was associated with the development of postoperative HF in intermediate-risk surgical patients with preserved EF.

## Introduction

Heart failure (HF) is associated with postoperative mortality among patients undergoing elective non-cardiac surgery [1]. It is related to left ventricular (LV) reduced systolic function, but by recent report, it is not always dependent on systolic function [1, 2]. Among patients undergoing elective non-cardiac surgery, 7.9% patients have already had a clinical HF with and without symptoms, and in those patients, 60% had documented preserved ejection fraction (EF) [1]. Nearly half of all HF patients are HF with preserved EF (HFpEF), and their morbidity and mortality are similar to those with reduced EF [1, 3]. HFpEF patients are found to have LV diastolic dysfunction, older age, and more likely to have comorbidities such as hypertension (HT), diabetes mellitus (DM), chronic obstructive pulmonary disease (COPD), chronic kidney disease (CKD), and atrial fibrillation (AF) [1, 3]. Many elderly patients who need surgery often have these comorbidities [4], which is likely to have diastolic dysfunction with preserved EF.

The LV diastolic dysfunction is also reported to be an independent risk factor for adverse cardiovascular morbidities and mortalities in high-risk surgical patients including cardiovascular surgery and sepsis patients [5–8]. A study reported that diastolic dysfunction is also a predictor of pulmonary edema and cardiovascular complication in elderly patients undergoing low or intermediate risk non-cardiac surgery [9].

As there were only a few investigations among intermediate-risk surgical patients, this study aimed to investigate the incidence of postoperative HF in intermediate-risk (gastrointestinal, hepatobiliary, or pancreatic) surgical patients who had preoperative diastolic dysfunction with preserved EF. We hypothesized that the presence of preoperative LV diastolic dysfunction would increase the development of postoperative HF among intermediate-risk surgical patients with preserved EF.

## Methods

The study was approved by the institutional review board approval (No. 2018M083) of Fukuoka University, Fukuoka, Japan, and a waiver of informed consent was obtained.

Consecutive patients who underwent gastrointestinal, hepatobiliary, or pancreatic surgery under general anesthesia between January 2016 and December 2018 were retrospectively evaluated. Patients who underwent low-risk surgery, who did not examine transthoracic echocardiography before surgery, who have incomplete echocardiography data, or reduced EF (EF < 50%) were excluded.

Preoperative transthoracic echocardiographic examination with tissue Doppler measurements was performed. To assess LV systolic function, EF was calculated using the biplane Simpson’s technique. To assess LV diastolic function, we used one of the parameters of LV diastolic function: the ratio of LV early diastolic filling velocity to the septal peak diastolic velocity of the mitral medial annulus (*E*/*e*’) measured by transthoracic echocardiography. Patients with normal (preserved) LV systolic function (EF ≥ 50%) were divided into three groups as follows: *E*/*e*’ ≥ 15, *E*/*e*’ between 8 and 15, and *E*/*e*’ < 8 [10].

Postoperative HF was defined as clinical and radiological evidence of pulmonary edema that required diuretics and/or a vasodilator, and low SpO_2_ less than 93% with oxygen therapy with/without mechanical ventilation up to after 7 days after surgery. Postoperative pulmonary edema and congestion were based on the following findings: perivascular cuffing, Kerley’s line, peribronchial cuffing, vanishing tumor, butterfly shadow, and/or dull of costophrenic angle, and these findings were confirmed by two independent physicians. Patients in whom HF was present before surgery were excluded from the criteria of postoperative HF.

Clinical and laboratory data were collected retrospectively from the anesthetic and medical records. Ischemic heart disease (IHD) was defined as clinical history of myocardial infarction or stable angina pectoris, and HT was defined as a patient with more than 140/90 mmHg or anti-hypertensive therapy. DM was defined as a fasting blood sugar more than 126 mg/dl or HbA1c more than 6.5%. Dyslipidemia was defined as triglyceride more than 150 mg/dl or low-density lipoprotein (LDL) cholesterol more than 140 mg/dl, or a patient who received hyperlipidemia therapy. COPD was defined as spirometry FEV_1.0_/FVC less than 70% or a patient who was diagnosed with emphysema. CKD was defined as estimated glomerular filtration rate (eGFR) less than 60 ml/min/1.73 m^2^. The primary endpoint of this study was the incidence of postoperative HF in intermediate-risk surgical patients who had preoperative diastolic dysfunction with preserved EF.

### Statistical analysis

Data are presented as mean ± standard deviation. Incidences are presented as percentage, and 95% confidence intervals (95CI) are presented for outcomes. Chi-squared test or analysis of variance was used to compare categories. If *P* value was < 0.05, chi-squared test or unpaired *t* test with Welch’s correction was used to compare each group. Multivariate logistic regression was used to adjust for the effects of other factors.

An ad hoc power analysis was performed to determine the statistical power of this study. Assuming the rate of HF development is 1% in normal patients and 10% in patients with cardiac dysfunction, based on a previous study that reported that 15–20% of high-risk surgical patients developed postoperative HF [7, 11], with an α error of 0.05 and a 1–*β* error of 0.8, a total sample size of 244 patients (122 patients per group) would detect a difference in our primary outcome between groups based on a chi-squared test.

Statistical analyses were performed using GraphPad Prism (version 6; GraphPad Software Inc., San Diego, CA). A significance value of 0.05 was used for each test.

## Results

**Fig. 1:**
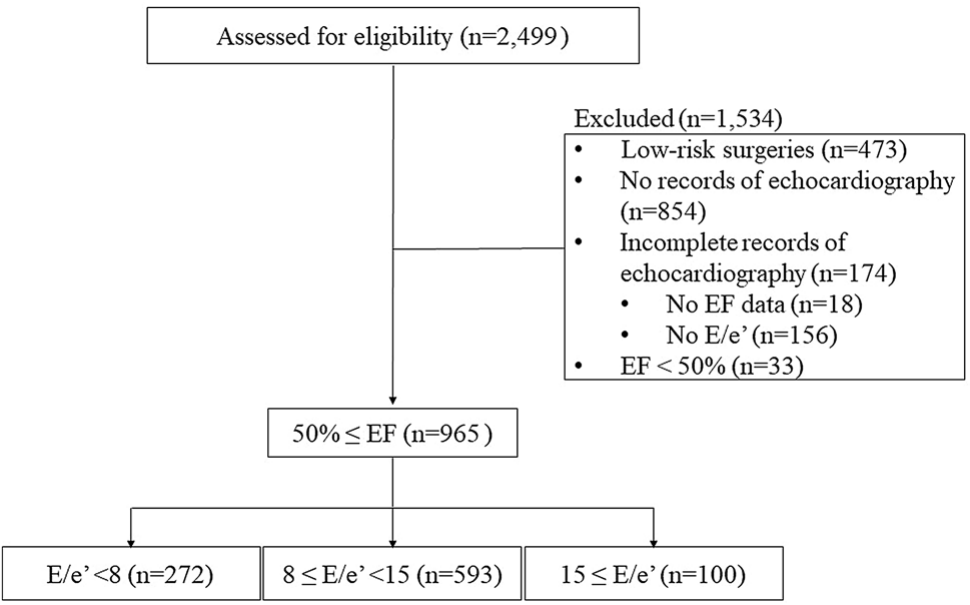
Study inclusion/exclusion flow diagram. A total of 2499 patients underwent general surgery under general anesthesia during the study period. After excluding patients who underwent low-risk surgery (*n* = 473), who were not examined by echocardiography (*n* = 854), who had incomplete echocardiography data (*n* = 174), or who with reduced EF (EF < 50%) (*n* = 33) the remaining 965 patients were included in the final analysis. *EF* left ventricular ejection fraction, *E*/*e*’ ratio of LV early diastolic filling velocity (*E*) to the peak diastolic velocity of mitral medial annulus (*e*’)

A total of 2499 patients underwent gastrointestinal, hepatobiliary, or pancreatic surgery under general anesthesia during the study period. After excluding patients who underwent low-risk surgery (*n* = 473), with no or incomplete echocardiographic data (*n* = 1028), and with reduced EF (EF < 50%) (*n* = 33), 965 patients were included in the final analysis (Fig. 1). There were 272, 593, and 100 patients with *E*/*e*’ < 8, *E*/*e*’ between 8 and 15, and *E*/*e*’ ≥ 15 with preserved EF (EF > 50%), respectively (Fig. 1).

Patients’ characteristics are shown in Table 1. Among patients with preserved EF, those who had *E*/*e*’ between 8 and 15 and *E*/*e*’ ≥ 15 had significantly higher proportion of elderly patients (*P* < 0.01), and who had *E*/*e*’ ≥ 15 had significantly higher proportion of female patients than those who had *E*/*e*’ < 8 (*P* < 0.01). The rates of IHD, HT, DM, dyslipidemia, AF, COPD, CKD, and hemodialysis were higher in patients with *E*/*e*’ ≥ 15 than those who had *E*/*e*’ < 8 (*P* < 0.01). The rates of HT, DM, dyslipidemia, and COPD were higher in patients with *E*/*e*’ between 8 and 15 than those who had *E*/*e*’ < 8 (*P* < 0.01).

**Table 1:** Patients’ characteristics

	*E*/*e*’ < 8 (*n* = 272)	8 ≤ *E*/*e*’ < 15 (*n* = 593)	15 ≤ *E*/*e*’ (*n* = 100)
Age, mean ± SD, years (*P* value)	64 ± 14	70 ± 11* (< 0.0001)	78 ± 7* (< 0.0001)
Female, no. (%) (*P* value)	98 (36)	245 (38) (0.14)	54 (54)* (0.0018)
BMI, mean ± SD, kg/m^2^ (*P* value)	22.1 ± 2.2	22.6 ± 1.7 (0.055)	22.4 ± 3.2 (0.44)
ASA-PS (I/II/III/IV)	31/223/18/0	42*/472/78*/1	0*/73/15*/2*
(*P* value)			
(I)		(0.034)	(0.0004)
(II)		(0.41)	(0.057)
(III)		(0.0045)	(0.012)
(IV)		(0.50)	(0.019)
IHD, no. (%) (*P* value)	17 (6.3)	61 (10) (0.075)	23 (23)* (< 0.0001)
Hypertension, no. (%) (*P* value)	102 (38)	322 (54)* (< 0.0001)	70 (70)* (< 0.0001)
Diabetes mellitus, no. (%) (*P* value)	42 (24)	158 (27)* (0.006)	28 (28)* (0.0003)
Dyslipidemia, no. (%) (*P* value)	42 (24)	153 (26)* (0.0007)	35 (35)* (0.006)
Atrial fibrillation, no. (%) (*P* value)	8 (2.9)	26 (4.4) (0.31)	8 (8)* (0.033)
COPD, No. (%) (*P* value)	53 (19)	177 (29)* (0.0014)	41 (41)* (< 0.0001)
CKD, no. (%) (*P* value)	75 (28)	202 (34) (0.056)	59 (59)* (< 0.0001)
Hemodialysis, no. (%) (*P* value)	0 (0)	2 (0.3) (0.34)	5 (5)* (< 0.0001)

*E*/*e*′ ratio of LV early diastolic filling velocity (*E*) to the peak velocity of mitral medial annulus (*e*’), *BMI* body mass index, *ASA-PS* American Society of Anesthesiologists Physical Status, *IHD* ischemic heart disease, *No* number, *COPD* chronic obstructive pulmonary disease, *CKD* chronic kidney disease, *SD* standard deviation, Welch’s *t* test and Chi-square test were used for statistical differences between groups

^*^p < 0.05 compared with *E*/*e*’ < 8

The type of surgery and category of *E*/*e*’ are shown in Table 2. Patient who underwent cholecystectomy had a significantly higher proportion of patients with *E*/*e*’ ≥ 15 than patients with *E*/*e*’ < 8 (*P* = 0.0047) (Table 2).

**Table 2:** Type of surgery

	Total	*E*/*e*’ < 8 (*n* = 272)	8 ≤ *E*/*e*’ < 15 (*n* = 593)	15 ≤ *E*/*e*’ (*n* = 100)
Esophagus, no. (%) (*P* value)	47	16 (5.9)	28 (4.7) (0.47)	3 (3.0) (0.26)
Upper GI, no. (%) (*P* value)	273	80 (29)	169 (28) (0.78)	24 (24) (0.30)
Lower GI, no. (%) (*P* value)	372	108 (40)	223 (38) (0.56)	41 (41) (0.82)
Liver, no. (%) (*P* value)	89	26 (9.6)	56 (9.4) (0.96)	7 (7.0) (0.44)
Cholecystectomy, no. (%) (*P* value)	130	27 (9.9)	82 (14) (0.11)	21 (21)* (0.0047)
Pancreas, no. (%) (*P* value)	52	16 (5.9)	32 (5.4) (0.77)	4 (4.0) (0.48)

*E*/*e*′ ratio of LV early diastolic filling velocity (*E*) to the peak velocity of mitral medial annulus (*e*’), *No* number, *GI* gastrointestinal, Chi-squared test was used to compare categories

^*^p < 0.05 compared with *E*/*e*’ < 8

Preoperative echocardiographic parameters are listed in Table 3. EF was not significantly different among patients with preserved EF. *E*/*e*’ in patients with *E*/*e*’ between 8 and 15, and *E*/*e*’ ≥ 15 were significantly higher than that in patients with *E*/*e*’ < 8. The LV wall (interventricular septum thickness, posterior wall thickness) was thicker and LAD was larger in patients with *E*/*e*’ between 8 and 15 and *E*/*e*’ ≥ 15 than in patients with *E*/*e*’ < 8.

**Table 3:** Preoperative transthoracic echocardiographic variables

	*E*/*e*’ < 8 (*n* = 272)	8 ≤ *E*/*e*’ < 15 (*n* = 593)	15 ≤ *E*/*e*’ (*n* = 100)
EF (%) (*P* value)	67 ± 5.9	68 ± 5.7 (0.49)	67.4 ± 6.3 (0.62)
LAD (mm) (*P* value)	33.9 ± 6.4	35.5 ± 6.3* (0.014)	38.1 ± 7.4* (0.0002)
LVDd (mm) (*P* value)	44.3 ± 5.1	44.0 ± 5.3 (0.53)	43.8 ± 6.2 (0.50)
LVDs (mm) (*P* value)	27.5 ± 3.9	27.4 ± 4.0 (0.73)	27.4 ± 4.8 (0.73)
IVST (mm) (*P* value)	9.1 ± 1.4	9.7 ± 3.4* (0.0002)	9.9 ± 1.7* (< 0.0001)
PWT (mm) (*P* value)	9.4 ± 1.3	9.7 ± 1.3* (0.0017)	10.0 ± 1.4* (0.0001)
*E*/A (*P* value)	0.89 ± 0.68	0.85 ± 0.36 (0.32)	0.87 ± 0.33 (0.67)
*E*/*e*' (*P* value)	6.5 ± 1.1	10.7 ± 1.9* (< 0.0001)	19.4 ± 5.4* (< 0.0001)

Data are presented as mean ± standard deviation. Welch’s *t* test as used for differences in mean value

*E*/*e*′ ratio of LV early diastolic filling velocity (*E*) to the peak velocity of mitral medial annulus (*e*’), *EF* ejection fraction, *LAD* left anterior descending artery, *LVDd* left ventricular diastolic dimension, *LVDs* left ventricular systolic dimension, *IVST* interventricular septal thickness, *PWT* posterior wall thickness, *E*/*A* mean *E* and late filling (*A*) ratio

^*^p < 0.05 compared with *E*/*e*’ < 8

**Fig. 2:**
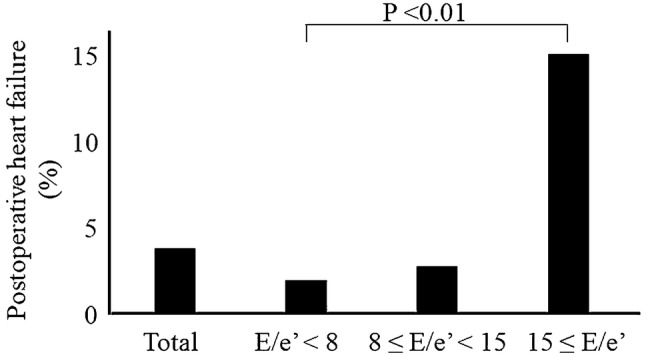
Incidence of postoperative heart failure. A total of 36 patients (3.7%) with preserved EF developed postoperative HF. The incidence of postoperative HF was stratified according to the *E*/*e*’ ratio, and the proportion of HF patients with *E*/*e*’ < 8, 8–15, and ≥ 15 was 1.2%, 1.9%, and 11.1%, respectively (*P* < 0.01). *E*/*e*’ ratio of LV early diastolic filling velocity (*E*) to the peak diastolic velocity of mitral medial annulus (*e*’)

A total of 36 patients (3.7%) with preserved EF developed postoperative HF. The incidence of postoperative HF is shown in Fig. 2. In these patients with preserved EF, the incidence of postoperative HF was stratified according to the *E*/*e*’ ratio, and the proportion of HF patients with *E*/*e*’ < 8, 8–15, and ≥ 15 was 1.8%, 2.7%, and 15%, respectively (*P* < 0.01). In 36 patients who developed postoperative HF, there was no significant difference among the type of surgery: esophageal surgery (4.2%), upper gastrointestinal surgery (3.6%), lower gastrointestinal surgery (4.6%), liver surgery (5.6%), cholecystectomy (1.5%), and pancreas surgery (0%) (*P* = 0.36). These patients who developed HF were treated with a vasodilator and/or diuretics and oxygen, and no patients needed mechanical ventilation nor died.

Table 4 presents intraoperative parameters. The duration of operation and anesthesia were significantly shorter in patients with *E*/*e*’ ≥ 15 than in patients with *E*/*e*’ < 8. (*P* < 0.01). Urine volume in patients with *E*/*e*’ between 8 and 15, and *E*/*e*’ ≥ 15 were significantly less than in *E*/*e*’ < 8. (*P* < 0.01) However, in–out balance was significantly less in patients with *E*/*e*’ ≥ 15 than in patients with *E*/*e*’ < 8 (*P* < 0.01).

**Table 4:** Intraoperative data

	*E*/*e*’ < 8 (*n* = 272)	8 ≤ *E*/*e*’ < 15 (*n* = 593)	15 ≤ *E*/*e*’ (*n* = 100)
Duration of operation, mean [95CI], minutes (*P* value)	359 [336–382]	350 [335–365] (0.54)	289* [256–322] (0.0009)
Duration of anesthesia, mean [95CI], minutes (*P* value)	465 [440–480]	457 [440–474] (0.62)	391* [355–427] (0.0012)
Urine volume, mean [95CI], ml (*P* value)	546 [491–601]	452* [420–484] (0.0038)	359* [289–429] (< 0.0001)
Blood loss, median [95CI], g (*P* value)	145 [111–178]	155 [125–185] (0.65)	113 [56–1169] (0.34)
In–out balance, mean [95CI], ml (*P* value)	1973 [1827–2119]	1979 [1817–2141] (0.96)	1456* [1275–1637] (< 0.0001)

Welch’s *t *test was used for differences in mean value

*E*/*e*′ ratio of LV early diastolic filling velocity (*E*) to the peak velocity of mitral medial annulus (*e*’), *95CI* 95% confidence intervals

^*^p < 0.05 compared with *E*/*e*’ < 8

Table 5 shows the results of the exact logistic regression. Adjusted odds ratio (OR) of *E*/*e*’ ≥ 15 was 9.31 (95%CI 3.29, 25.4, *P* < 0.01), and age, HT, and dyslipidemia were also increased (*P* < 0.05).

**Table 5:** Odds ratios and 95% confidence interval of post-operative heart failure

	Adjusted OR [95CI]	*P* value
8 ≤ *E*/*e*’ < 15	1.49 [0.54–4.10]	0.44
15 ≤ *E*/*e*’	9.31 [3.29–25.4]	0.000026
EF	1.04 [0.99–1.10]	0.15
Age	1.05 [1.01–1.09]	0.0058
In–out balance	1.00 [1.00–1.00]	0.85
IHD	2.24 [0.97–5.14]	0.059
Hypertension	2.95 [1.23–7.06]	0.015
Diabetes mellitus	1.15 [0.54–2.42]	0.72
Dyslipidemia	2.20 [1.05–4.60]	0.037
Atrial fibrillation	2.91 [0.99–8.57]	0.052
COPD	0.73 [0.33– 1.63]	0.44
CVA	1.52 [0.64–3.59]	0.34

Multivariate logistic regression was used to adjust for the effects of factors. *OR* odds ratios, *95CI* 95% confidence intervals, *E*/*e*′ ratio of LV early diastolic filling velocity (*E*) to the peak velocity of mitral medial annulus (*e*’), *IHD* ischemic heart disease, *COPD* chronic obstructive pulmonary disease, *CVA* cerebrovascular accident

## Discussion

With the above results, we found that elevated *E*/*e*’ (≥ 15) is a predictor of the development of postoperative HF in intermediate-risk (gastrointestinal, hepatobiliary, or pancreatic) surgical patients with preserved EF.

Postoperative HF has been reported to develop in 9.3% of high-risk patients and in 5.3% of low- or intermediate-risk elderly patients who underwent non-cardiac surgery [9, 11]. In our patients who underwent intermediate-risk (gastrointestinal, hepatobiliary, or pancreatic) surgery, HF occurred in 3.7% of patients with preserved EF. The rate of postoperative HF development in our study was similar to previous intermediate-risk surgery [9], which are also reasonable compared to high-risk patients because the reported 30-day mortality rate and rate of major adverse cardiac event was 1–5% in low- or intermediate-risk surgery and > 5% in high-risk surgery [12].

LV diastolic dysfunction has been associated with higher rates of morbidity and mortality after cardiac surgery [6, 13–15], which was also reported in a systemic review and meta-analysis after non-cardiac surgeries [5]. Moreover, the grade of the diastolic dysfunction is important for postoperative outcome, and only *E*/*e*’ ≥ 15 is found to be a risk factor [6]. However, most reports focused on high-risk non-cardiac surgery such as vascular surgery [7, 16], intermediate-risk surgery such as a carotid endarterectomy [17, 18], kidney transplantation [19–21], or lung transplantation [22, 23]. There are only a few reports on intermediate-risk non-cardiac surgical patients, and these reported the patients with cardiovascular disease and elderly patients [9, 17]. In our study, patients have undergone intermediate-risk surgery such as gastrointestinal, hepatobiliary, pancreatic, and cholecystectomy, and we included patients without history of cardiovascular disease and young patients. In these intermediate-risk surgical patients, our results clearly showed that *E*/*e*’ ≥ 15, age, HT, and dyslipidemia were predictors for postoperative HF. Especially, the OR of *E*/*e*’ ≥ 15 was high (9.31), which indicated that *E*/*e*’ ≥ 15 was a strong predictor for postoperative HF in patients with preserved EF.

In our study, patients with elevated *E*/*e*’ (*E*/*e*’ ≥ 15) were elderly and have HT, IHD, AF, DM, CKD, anemia (data not shown), dyslipidemia, and COPD. This result is similar to patients with HFpEF who are highly likely to have IHD, HT, AF, and secondary cardiomyopathy due to DM, CKD, liver disease, and metabolic syndrome [24]. Most surgical patients are commonly at an advanced age, and they often have several comorbidities such as HT, IHD, DM, AF, CKD, anemia, dyslipidemia, and COPD [4]. Therefore, we should know that patients with these comorbidities are at a high risk for diastolic dysfunction, which could contribute to the development of postoperative HF.

To avoid developing postoperative HF and pulmonary edema, standard clinical practice of fluid therapy is to avoid the overloading of fluid during perioperative period. In our study, patients with *E*/*e*’ ≥ 15 had significantly lower intraoperative in–out balance. This suggests that each anesthesiologist recognized the elevated *E*/*e*’, and patients had limited fluid therapy during operation. Nevertheless, they developed postoperative HF. It may indicate that it is important to manage the loading condition of heart in not only intraoperative but also postoperative period.

This study has several limitations. First, this was a retrospective study and treatment strategy was not controlled. Preoperative cardiac echocardiography was ordered by either the surgeon or the anesthesiologist. The attending anesthesiologist could control anesthetic technique and fluid volume by knowing the impaired LV diastolic function. In fact, patients with *E*/*e*’ ≥ 15 had significantly less in–out balance. Nevertheless, postoperative HF was developed in patients with *E*/*e*’ > 15. Second, we categorized patients into three groups only by *E*/*e*’. However, in the guideline, the algorithm for the diagnosis of diastolic dysfunction in patients with normal EF is now based on four categories, namely, mean *E*/*e*’ > 14, septal or lateral *e*’ velocity, TR velocity, and left atrial volume index [4]. Therefore, our *E*/*e*’ ≥ 15 category may overestimate diastolic dysfunction. Third, preoperative transthoracic echocardiography was performed by several sonographers, and findings were evaluated by several cardiologists, which could affect inter-observer variability of echocardiographic parameters. Fourth, each group should have more than 122 patients; however, there were only 100 patients with *E*/*E*’ > 15 with preserved EF; thus, power was less. Fifth, we defined HF as a symptom of low SpO_2_ less than 93%, even if oxygen therapy was used, and pulmonary congestion and cardiomegaly on chest X-ray, but this definition could not detect all patients who developed HF.

In conclusion, postoperative HF developed in 3.7% of the general surgical patients with preserved LV systolic function. The incidence of postoperative HF was 15% in patients who had *E*/*e*’ ≥ 15 with preserved LV systolic function, more than in patients with E/e’ < 8 or between 8 and 15. These findings suggest that the presence of preoperative elevated *E*/*e*’ (≥ 15) is associated with the development of postoperative HF in intermediate-risk surgical patients with preserved EF.

## References

[1] Lerman BJ, Popat RA, Assimes TL, Heidenreich PA, Wren SM (2019). Association of left ventricular ejection fraction and symptoms with mortality after elective noncardiac surgery among patients with heart failure. JAMA.

[2] Dalen M, Lund LH, Ivert T, Holzmann MJ, Sartipy U (2016). Survival after coronary artery bypass grafting in patients with preoperative heart failure and preserved vs reduced ejection fraction. JAMA Cardiol..

[3] Tsutsui H, Tsuchihashi-Makaya M, Kinugawa S (2010). Clinical characteristics and outcomes of heart failure with preserved ejection fraction: lessons from epidemiological studies. J Cardiol..

[4] Nagueh SF, Smiseth OA, Appleton CP, Byrd BF, Dokainish H, Edvardsen T, Flachskampf FA, Gillebert TC, Klein AL, Lancellotti P, Marino P, Oh JK, Popescu BA, Waggoner AD (2016). Recommendations for the evaluation of left ventricular diastolic function by echocardiography: an update from the American Society of Echocardiography and the European Association of Cardiovascular Imaging. J Am Soc Echocardiogr..

[5] Fayad A, Ansari MT, Yang H, Ruddy T, Wells GA (2016). Perioperative diastolic dysfunction in patients undergoing noncardiac surgery is an independent risk factor for cardiovascular events: a systematic review and meta-analysis. Anesthesiology.

[6] Kaw R, Hernandez AV, Pasupuleti V, Deshpande A, Nagarajan V, Bueno H, Coleman CI, Ioannidis JP, Bhatt DL, Blackstone EH (2016). Effect of diastolic dysfunction on postoperative outcomes after cardiovascular surgery: a systematic review and meta-analysis. J Thorac Cardiovasc Surg..

[7] Matyal R, Hess PE, Subramaniam B, Mitchell J, Panzica PJ, Pomposelli F, Mahmood F (2009). Perioperative diastolic dysfunction during vascular surgery and its association with postoperative outcome. J Vasc Surg..

[8] Rolando G, Espinoza ED, Avid E, Welsh S, Pozo JD, Vazquez AR, Arzani Y, Masevicius FD, Dubin A (2015). Prognostic value of ventricular diastolic dysfunction in patients with severe sepsis and septic shock. Rev Bras Ter Intensiva..

[9] Cho DH, Park SM, Kim MN, Kim SA, Lim H, Shim WJ (2014). Presence of preoperative diastolic dysfunction predicts postoperative pulmonary edema and cardiovascular complications in patients undergoing noncardiac surgery. Echocardiography..

[10] Paulus WJ, Tschope C, Sanderson JE, Rusconi C, Flachskampf FA, Rademakers FE, Marino P, Smiseth OA, De Keulenaer G, Leite-Moreira AF, Borbely A, Edes I, Handoko ML, Heymans S, Pezzali N, Pieske B, Dickstein K, Fraser AG, Brutsaert DL (2007). How to diagnose diastolic heart failure: a consensus statement on the diagnosis of heart failure with normal left ventricular ejection fraction by the Heart Failure and Echocardiography Associations of the European Society of Cardiology. Eur Heart J..

[11] Arulkumaran N, Corredor C, Hamilton MA, Ball J, Grounds RM, Rhodes A, Cecconi M (2014). Cardiac complications associated with goal-directed therapy in high-risk surgical patients: a meta-analysis. Br J Anaesth..

[12] Fleisher LA, Beckman JA, Brown KA, Calkins H, Chaikof E, Fleischmann KE, Freeman WK, Froehlich JB, Kasper EK, Kersten JR, Riegel B, Robb JF, Smith SC, Jacobs AK, Adams CD, Anderson JL, Antman EM, Buller CE, Creager MA, Ettinger SM, Faxon DP, Fuster V, Halperin JL, Hiratzka LF, Hunt SA, Lytle BW, Nishimura R, Ornato JP, Page RL, Tarkington LG, Yancy CW (2007). ACC/AHA 2007 guidelines on perioperative cardiovascular evaluation and care for noncardiac surgery: a report of the American College of Cardiology/American Heart Association Task Force on Practice Guidelines (Writing Committee to Revise the 2002 Guidelines on Perioperative Cardiovascular Evaluation for Noncardiac Surgery): developed in collaboration with the American Society of Echocardiography, American Society of Nuclear Cardiology, Heart Rhythm Society, Society of Cardiovascular Anesthesiologists, Society for Cardiovascular Angiography and Interventions, Society for Vascular Medicine and Biology, and Society for Vascular Surgery. Circulation.

[13] Chang SA, Park PW, Sung K, Lee SC, Park SW, Lee YT, Oh JK (2010). Noninvasive estimate of left ventricular filling pressure correlated with early and midterm postoperative cardiovascular events after isolated aortic valve replacement in patients with severe aortic stenosis. J Thorac Cardiovasc Surg..

[14] Lee EH, Yun SC, Chin JH, Choi DK, Son HJ, Kim WC, Choi SS, Song JG, Hahm KD, Sim JY, Choi IC (2012). Prognostic implications of preoperative *E*/*e*' ratio in patients with off-pump coronary artery surgery. Anesthesiology.

[15] Metkus TS, Suarez-Pierre A, Crawford TC, Lawton JS, Goeddel L, Dodd OJ, Mukherjee M, Abraham TP, Whitman GJ (2018). Diastolic dysfunction is common and predicts outcome after cardiac surgery. J Cardiothorac Surg..

[16] Flu WJ, van Kuijk JP, Hoeks SE, Kuiper R, Schouten O, Goei D, Elhendy A, Verhagen HJ, Thomson IR, Bax JJ, Fleisher LA, Poldermans D (2010). Prognostic implications of asymptomatic left ventricular dysfunction in patients undergoing vascular surgery. Anesthesiology.

[17] Saito S, Takagi A, Kurokawa F, Ashihara K, Hagiwara N (2012). Usefulness of tissue Doppler echocardiography to predict perioperative cardiac events in patients undergoing noncardiac surgery. Heart Vessels..

[18] Shigematsu K, Iwashita K, Mimata R, Owaki R, Totoki T, Gohara A, Okawa J, Higashi M, Yamaura K (2019). Preoperative left ventricular diastolic dysfunction is associated with pulmonary edema after carotid endarterectomy. Neurol Med Chir (Tokyo)..

[19] Canet E, Osman D, Lambert J, Guitton C, Heng AE, Argaud L, Klouche K, Mourad G, Legendre C, Timsit JF, Rondeau E, Hourmant M, Durrbach A, Glotz D, Souweine B, Schlemmer B, Azoulay E (2011). Acute respiratory failure in kidney transplant recipients: a multicenter study. Crit Care..

[20] Higashi M, Yamaura K, Ikeda M, Shimauchi T, Saiki H, Hoka S (2013). Diastolic dysfunction of the left ventricle is associated with pulmonary edema after renal transplantation. Acta Anaesthesiol Scand..

[21] Kim EJ, Chang S, Kim SY, Huh KH, Kang S, Choi YS (2016). Predictive value of echocardiographic abnormalities and the impact of diastolic dysfunction on in-hospital major cardiovascular complications after living donor kidney transplantation. Int J Med Sci..

[22] Gupta S, Torres F, Bollineni S, Mohanka M, Kaza V (2015). Left ventricular dysfunction after lung transplantation for pulmonary arterial hypertension. Transplant Proc..

[23] Avriel A, Klement AH, Johnson SR, de Perrot M, Granton J (2017). Impact of left ventricular diastolic dysfunction on lung transplantation outcome in patients with pulmonary arterial hypertension. Am J Transplant..

[24] Miyagishima K, Hiramitsu S, Kimura H, Mori K, Ueda T, Kato S, Kato Y, Ishikawa S, Iwase M, Morimoto S, Hishida H, Ozaki Y (2009). Long term prognosis of chronic heart failure: reduced vs preserved left ventricular ejection fraction. Circ J..

